# Editorial: Phosphorus starvation in plants

**DOI:** 10.3389/fpls.2023.1211439

**Published:** 2023-05-26

**Authors:** Rahul Kumar, Umakanta Ngangkham, Siti Nor Akmar Abdullah

**Affiliations:** ^1^ Department of Plant Sciences, University of Hyderabad, Hyderabad, Telangana, India; ^2^ ICAR Research Complex for NEH Region Manipur Centre, Manipur, India; ^3^ Faculty of Agriculture, Universiti Putra Malaysia, Serdang, Selangor, Malaysia

**Keywords:** phosphorus starvation response, Pi use efficiency, Pi acquisition efficiency, Pi recycling, transcriptome (RNA-seq), plant–fungal symbiotic interactions, mycorrhiza

Phosphorus (P) is an essential macronutrient critical for plant growth and development. Its limited availability often affects plant performance and determines crop productivity. P is assimilated by plants in the form of its inorganic form, mainly orthophosphate (Pi). Chemical Pi fertilizers are applied to overcome soil Pi deficiency and augment plant produce. The main source of P fertilizer is rock phosphate, a finite and non-renewable resource. However, the assimilation of only 20-30% of the total exogenously applied Pi input makes the entire fertigation process inefficient. The unassimilated surplus Pi comes with environmental consequences. Therefore, the experts have recommended the identification or generation of Pi-acquisition efficient or Pi-use-efficient high-yielding crop germplasm to cut down overall Pi usage in the farming system. While current Pi fertilizers production and usage are in balance, the demand for this resource is expected to outperform the supply soon, which would disrupt Pi fertilizers distribution chains and food production. Therefore, immediate solutions need to be found to cut down its usage in agriculture and prolong the life of natural Pi reserves. In this special issue, seven articles (five original research and two review articles) further augment the current knowledge of Pi starvation responses in plants. Luo et al., in an original research article, catalogs Pi starvation responses at physiological and molecular levels in elephant grass leaves and roots. The authors report higher acid phosphatase and Pi-use-efficiency in Pi-deficient elephant grass plants, which corroborates with the observations reported in other plant species. The authors next perform RNA-sequencing and identify P starvation inducible (PSI) genes such as purple acid phosphatases and report an enrichment in genes encoding the inorganic pyrophosphate (PPi)-dependent bypass enzymes. Lastly, the authors perform metabolome profiling and report enhanced Pi-free lipids, phenylpropanoids, and flavonoids in the leaves and roots of Pi-deficient plants. Overall, this study identifies candidate unigenes involved in Pi-use-efficiency (PUE) and provides information useful for developing Pi-efficient elephant grass varieties. Roller et al. assess a large panel of maize (*Zea mays* L.) varieties, comprising almost 300 elite and double haploid accessions, for their growth and yield without P starter fertilization in multi-environment field trials and reports significant changes in early plant development but without much impact on grain yield, which remained unchanged in these tests. The authors propose using the observed genotypic variations to breed Pi-efficient maize cultivars to reduce Pi fertilizer in more sustainable agriculture. Yang et al. record the responses of ten different soybean varieties to low soil phosphorus availability and examine molecular regulatory mechanisms underlying root phosphorus acquisition strategies among varieties with different low-phosphorus tolerance using transcriptome sequencing. The analysis reveals two main phosphorus acquisition strategies—”outsourcing” and “do-it-yourself”— employed by soybean varieties under Pi deficient conditions. The “do-it-yourself” varieties rely on the increased root surface area and enhanced carboxylic acids secretion. The “outsourcing” varieties exhibit larger root diameters and enhanced mycorrhization potential. The authors found acetyl-CoA metabolism to be the dividing line between the two strategies and identified ERF1 and WRKY1 as key regulators of Pi starvation response (PSR). In another research article, Rong et al. analyze the effects of endophytic fungus *Serendipita indica* inoculation on growth, hormones concentrations, P levels, and mRNA abundance of two phosphate transporter (PT) genes in leaves of tea (*Camellia sinensis* L. cv. Fudingdabaicha) seedlings grown under two low Pi concentrations. *S. indica* inoculation was found to increase leaf P and promote plant growth. The hormone analysis suggests that the increased indoleacetic acid and cytokinins levels in the *S. indica* colonized tea plants could enhance Pi uptake by activating both PTs and promote growth. Mao et al. report changes in P fractions at different soil layers in response to different fertigation systems, such as cattle manure (M) or cattle manure and chemical fertilizer application (M+F) in open-field vegetable systems. As anticipated, the authors report higher concentrations of the soil P fractions in the top layers. They observe the highest vegetable yield with M+F fertigation system. Based on the results, the authors argue about the great potential of a combined manure-chemical fertilizer fertigation system to improve vegetable productivity with lower Pi input in open-field vegetable systems. In the minireview article, Yoshitake and Yoshimoto discuss the importance of Pi recycling to PSR and Pi homeostasis in Pi-deficient plants and summarize the current state Pi recycling pathways, including phosphoester degradation, nucleic acid degradation, membrane lipid remodeling, autophagy, and vacuolar transporter under Pi starvation. In the other review article, Chen et al. review the importance of root system architecture reprogramming, including traits such as the growth of primary root, lateral roots, root hairs, and cluster roots, to Pi acquisition efficiency in legumes. The authors summarize various strategies to improve root traits for enhancing PAE in legume crops. They also highlight the key functional genes and regulators of root trait reprogramming and discuss new opportunities for developing legume varieties with maximum PAE for regenerative agriculture. Overall, this is a unique collection of research articles where besides the basic research, original research articles, the impact of altered fertigation systems, endophytic fungi colonization, or the application of different varieties to breed Pi-uptake efficient varieties in multiple crops have been covered. In summary, the research covered in this special collection on P starvation in plants is anticipated to help researchers to develop methods for cutting down Pi usage in agriculture to make it more sustainable.

## Author contributions

RK wrote the original draft. The final draft was read and approved by all authors.

